# Prevalence and impact of cardiac injury on COVID‐19: A systematic review and meta‐analysis

**DOI:** 10.1002/clc.23540

**Published:** 2020-12-31

**Authors:** Linghua Fu, Xiao Liu, Yuhao Su, Jianyong Ma, Kui Hong

**Affiliations:** ^1^ Cardiology Department The Second Affiliated Hospital of Nanchang University Nanchang Jiangxi China; ^2^ Jiangxi Key Laboratory of Molecular Medicine Nanchang Jiangxi China

**Keywords:** cardiac injury, coronavirus disease 2019, death, intensive care unit

## Abstract

**Background:**

The exact prevalence and impact of cardiac injury in hospitalized patients with coronavirus disease 2019 (COVID‐19) is still controversial. Hence, we aim to investigate prevalence of cardiac injury and its impact on the outcomes in patients with COVID‐19.

**Hypothesis:**

Cardiac injury is common and associated with higher risk of death.

**Methods:**

We searched the Cochrane Library, PubMed, MedRxiv, and EMBASE databases from December 2019 to July 15, 2020 for studies that evaluated the prevalence and impact of cardiac injury on COVID‐19. This study has been registered with PROSPERO (International prospective register of systematic reviews)‐registration number‐CRD‐42020186120.

**Results:**

Twenty‐one studies including 6297 participants were identified. The proportions of cardiac injury were 22%, 28% among hospitalized patients with COVID‐19 or severe COVID‐19 patients, respectively. The incidences of cardiac injury in advance age (>60 years) (30%) was about two‐fold than young patients (<60 years) (15%) with COVID‐19. Severe cases (42%) have seven‐fold prevalence cardiac injury than in their non‐ severe counterparts (6%). Furthermore, cardiac injury is associated with an increased risk of all‐cause mortality in patients with COVID‐19 (OR 10.11, 95% CI 4.49–22.77). In patients with severe COVID‐19, cardiac injury is associated with an increased risk of all‐cause mortality (OR: 16.79, 95% CI: 5.52–51.02).

**Conclusions:**

This was the first meta‐analysis exploring the prevalence and impact of cardiac injury on COVID‐19. Cardiac injury is common in hospitalized patients and advanced age and severe COVID‐19 patients prone to experience more risk of cardiac injury. Furthermore, cardiac injury is associated with increased risk of all‐cause mortality.

## 1 INTRODUCTION

Coronavirus disease 2019 (COVID‐19) pandemic is an ongoing global public health emergency, caused by severe acute respiratory syndrome coronavirus 2 (SARS‐CoV‐2). As of July 11, 2020, there are 12 322 395 confirmed cases with 556 335 deaths reported in 216 countries.^1^ COVID‐19 is associated with an increased risk of acute respiratory distress syndrome and has adverse effects on other organ systems, including the heart, kidney, and liver.^2^ Cardiovascular disease is a common comorbidity in patients with COVID‐19.^3^ COVID‐19 also lead to many cardiovascular diseases, such as cardiac arrhythmias, myocardial infarction, cardiomyopathy, shock, and cardiac arrest.^4^

Cardiac injury is defined as the level of serum troponin with at least one value was above the 99th percentile upper reference according to latest guideline.^5^ Recently, a body of the literatures^4^, ^6^, ^7^, ^8^, ^9^, ^10^, ^11^, ^12^, ^13^, ^14^, ^15^, ^16^, ^17^, ^18^ shown that acute cardiac injury occurs in patients with COVID‐19. However, the reported prevalence of cardiac injury on COVID‐19 varies from one to another. In a cohort of 138 hospitalized patients with COVID‐19 from Wuhan,^4^ cardiac injury was reported in 11.2% of hospitalized patients and 22% of intensive care unit (ICU) patients. Another study from New York City has reported the existence of cardiac injury in 36% of patients hospitalized with COVID‐19.^14^ The exact prevalence of cardiac injury in hospitalized patients with COVID‐19 is still not clear.

Moreover, accumulating evidence^3^, ^4^, ^6^, ^7^, ^8^, ^9^, ^10^, ^11^, ^12^, ^13^, ^14^, ^15^, ^16^, ^17^, ^18^, ^19^, ^20^, ^21^, ^22^, ^23^, ^24^ supports the notion that acute cardiac injury leads to other poor outcomes patients with cardiac injury had a significantly higher mortality risk than those without cardiac injury. We therefore conducted a systematic review and meta‐analysis to investigate the reported prevalence and impact of cardiac injury on COVID‐19.

## 2 METHODS

This meta‐analysis was performed based on the Preferred Reporting Items for Systematic Reviews and Meta‐analyses (PRISMA) guidelines for studies that evaluate healthcare interventions (http://www.prisma‐ statement.org).^25^ This study has been registered with PROSPERO (International prospective register of systematic reviews)‐registration number‐CRD 42018090474. Severe COVID‐19 patients is defined as patients with COVID‐19 who are admitted to an ICU patients, while the non‐severe COVID‐19 patients is defined as hospital patients with COVID‐19 who are not admitted to an ICU. Ethical approval is not applicable for this study.

### 2.1 Literature search

We conducted computerized searches of the Cochrane Library, PubMed, MedRxiv ( https://www.medrxiv.org/), and Embase databases from December 2019 to July 2020 (Table S1 in Supplemental material). To identify studies involving relevant COVID‐19, we performed the search using the following terms: 2019‐novel coronavirus, SARS‐CoV‐2, COVID‐19, and 2019‐nCoV. To identify studies involving outcomes, we performed the search using the following terms: cardiac injury, myocardial injury, and cardiac troponin. The definition of cardiac injury was those adopted by the original studies (Table S2 in Supplemental material**).**

Two groups of keywords were combined using the Boolean operator “and.” No language restrictions were applied for the literature search. We also reviewed reference lists, relevant journals, and conference abstracts to identify relevant studies. Additionally, we searched ClinicalTrial.gov (https://www.clinicaltrials.gov/ ) to obtain information on studies that were terminated before being published.

### 2.2 Study selection

Studies were considered eligible if they (1) were cohort or nested case–control studies; (2) reported the prevalence of cardiac injury; (3) reported the association between cardiac injury and outcomes (e.g. all‐cause death) in this illness. Certain publication types (e.g., reviews, editorials, and animal studies) or studies with insufficient data were excluded from this analysis.

### 2.3 Data extraction and quality assessment

Two researchers (Linghua Fu and Yuhao Su) extracted the basic characteristics, including the first author, publication year, geographical location, study type, participants (sex, age, and sample size), and duration of follow‐up. For multiple reports using the same data, we included the articles with the longest follow‐up or the largest numbers of participants. The quality of all prevalence studies was independently assessed by the Joanna Briggs Institute critical appraisal checklist. In addition, we assessing the quality of included studies involved with the impact of cardiac injury by Newcastle–Ottawa quality scale (NOS), which entailed evaluations of the selection of cohorts, the comparability of cohorts, and the assessment of outcome. In this meta‐analysis, we defined studies with an NOS of ≥6 stars as moderate to high‐quality studies; studies which lower scores were defined as low‐quality studies.^26^, ^27^ Two researchers discussed the topics during several face‐to‐face and web‐based meetings.

### 2.4 Statistical analyses

Statistical analysis was performed using Stata 15.0 (StataCorp, College Station, Texas, https://www.stata.com/stata15/) and Review Manager Version 5.3 (the Nordic Cochrane Center, Rigshospitalet, Denmark; http://ims.cochrane.org/revman). Meta‐analyses involved with the prevalence of cardiac injury were performed by Stata software. The exact binomial (Clopper–Pearson) method was used to calculate 95% confidence intervals (CIs). Estimates were normalized using the Freeman–Tukey double arcsine transformation. We selected a random effects model to evaluate summary prevalence. The effect measurement estimate of mortality risk chosen was the odds ratio (ORs) in our study. Risk ratio and hazard ratio in other studies were considered to be OR, as OR was shown to be a more effective measure.^28^ If not available, the ORs were calculated by events and total numbers of patients in two groups. The natural logarithm of the OR (log [OR]) and its standard error (SElog [OR]) were calculated and then pooled using statistical software. Cochran's chi‐square test and I^2^ statistic were used to evaluate the heterogeneity among the included studies. I^2^ values of 25%, 50%, and 75% were considered to represent low, moderate, and high heterogeneity, respectively.^29^, ^30^ The statistical significance threshold was set at *p* < .05.

## 3 RESULTS

### 3.1 Study selection

We screened 811 potentially relevant articles in the Cochrane Library (*n* = 19), PubMed (*n* = 393), MedRxiv (*n* = 286), and Embase (*n* = 113) databases (Figure S1 in Supplemental Material). We excluded 91 studies after screening the titles and abstracts, and the full texts of the remaining 720 studies were reviewed. After a quick screening of the full‐text articles, 30 records were received full‐test review, and 9 excluded for the following reasons: (1) seven studies did not have relevant results, and (2) two studies^31^, ^32^ were based on same population. Finally, 21 studies^3^, ^4^, ^6^, ^7^, ^8^, ^9^, ^10^, ^11^, ^12^, ^13^, ^14^, ^15^, ^16^, ^17^, ^18^, ^19^, ^20^, ^21^, ^22^, ^23^, ^24^ were included in the present meta‐analysis.

### 3.2 Study characteristics and quality

The study characteristics were shown in Table 1. All included studies were published in 2020. The sample sizes of the included studies ranged from 16 to 2736, with a total of 6297 individuals. Most patients were male (56.1%). Among the 21 articles, 4^6^, ^8^, ^9^, ^14^ were conducted in the USA, 1^7^ was conducted in South Korea, and 16^3^, ^4^, ^10^, ^11^, ^12^, ^13^, ^15^, ^16^, ^17^, ^18^, ^19^, ^20^, ^21^, ^22^, ^23^, ^24^ were conducted in China. Majority of studies^3^, ^4^, ^6^, ^7^, ^8^, ^9^, ^11^, ^13^, ^14^, ^16^, ^17^, ^18^, ^19^, ^20^, ^21^, ^22^, ^23^, ^24^ were retrospective observational (case–control) studies, and three^10^, ^12^, ^15^ were prospective observational (cohort) studies.

**TABLE 1:** Clinical characteristics of the 21 included studies

Studies	Country	Study type	Number, N	Age, years	Male, *N*	Comorbidity, *N*						Cardiac injury, *N*
						HTN	Diabetes	CHD	CKD	Chronic lung diseases	Malignancy	
Zhou F^3^	China	Retrospective	191	56	119	58	36	15	2	6	2	33
Wang D^4^, ^31^	China	Retrospective	138	56	75	43	14	NA	4	4	10	10
Latif F^6^	USA	Retrospective	28	64	22	20	17	NA	10	10	5	13
Hong KS^7^	South Korea	Retrospective	98	55.4	38	30	9	NA	NA	3	4	11
Arentz M^8^	USA	Retrospective	21	70	11	NA	7	NA	10	7	NA	3
Aggarwal S^9^	USA	Retrospective	16	67	12	9	5	3	6	2	3	3
Yu Y^10^	China	Prospective	226	64	139	96	47	22	8	15	10	61
Yang F^11^	China	Retrospective	92	69.8	49	51	13	NA	2	1	4	31
Wei JF^12^	China	Prospective	101	49	54	21	14	5	NA	1	NA	16
Li X^13^	China	Retrospective	548	60	279	166	83	34	10	17	24	119
Lala A^14^	USA	Retrospective	2736	66.4	1630	1065	719	453	NA	273	195	985
Huang C^15^	China	Prospective	41	49	30	6	8	NA	NA	1	1	5
Han H^16^	China	Retrospective	273	58.86	97	NA	NA	NA	NA	NA	NA	27
Deng Q^17^	China	Retrospective	112	65	57	36	19	15	NA	4	NA	42
Yang R^18^	China	Retrospective	212	55.6	107	NA	NA	NA	NA	NA	NA	7
Yang X^19^	China	Retrospective	52	59.7	35	NA	9	NA	NA	4	2	12
Shi S^20^	China	Retrospective	416	64	205	127	60	44	14	12	9	82
Nie SF^21^	China	Retrospective	311	63	190	NA	NA	NA	NA	NA	NA	103
Guo T^22^	China	Retrospective	187	58.5	91	61	28	21	6	4	13	52
Deng Y^23^	China	Retrospective	225	54	124	58	26	NA	NA	25	8	66
Chen T^24^	China	Retrospective	274	62	171	93	47	NA	4	18	7	89

Abbreviations: CHD, coronary heart disease; CKD, chronic kidney disease; HTN, hypertension; N, number; NA, not available.

As shown in Supplementary Table 3, those 21 studies were critically appraised for quality using the Joanna Briggs Institute Critical Appraisal Checklist for reporting prevalence data. All studies were evaluated on the basis of data relevance and methodological rigor, and papers that met a minimum of six of the nine criteria.

The quality of the included studies we assessing the quality of included studies involved with the impact of cardiac injury was assessed and summarized in Supplementary Table 4. According to the NOS, all of the included studies were considered to be high quality, with a score range of 6–9.

### 3.3 The prevalence of cardiac injury in hospitalized patients with COVID‐19

**FIGURE 1:**
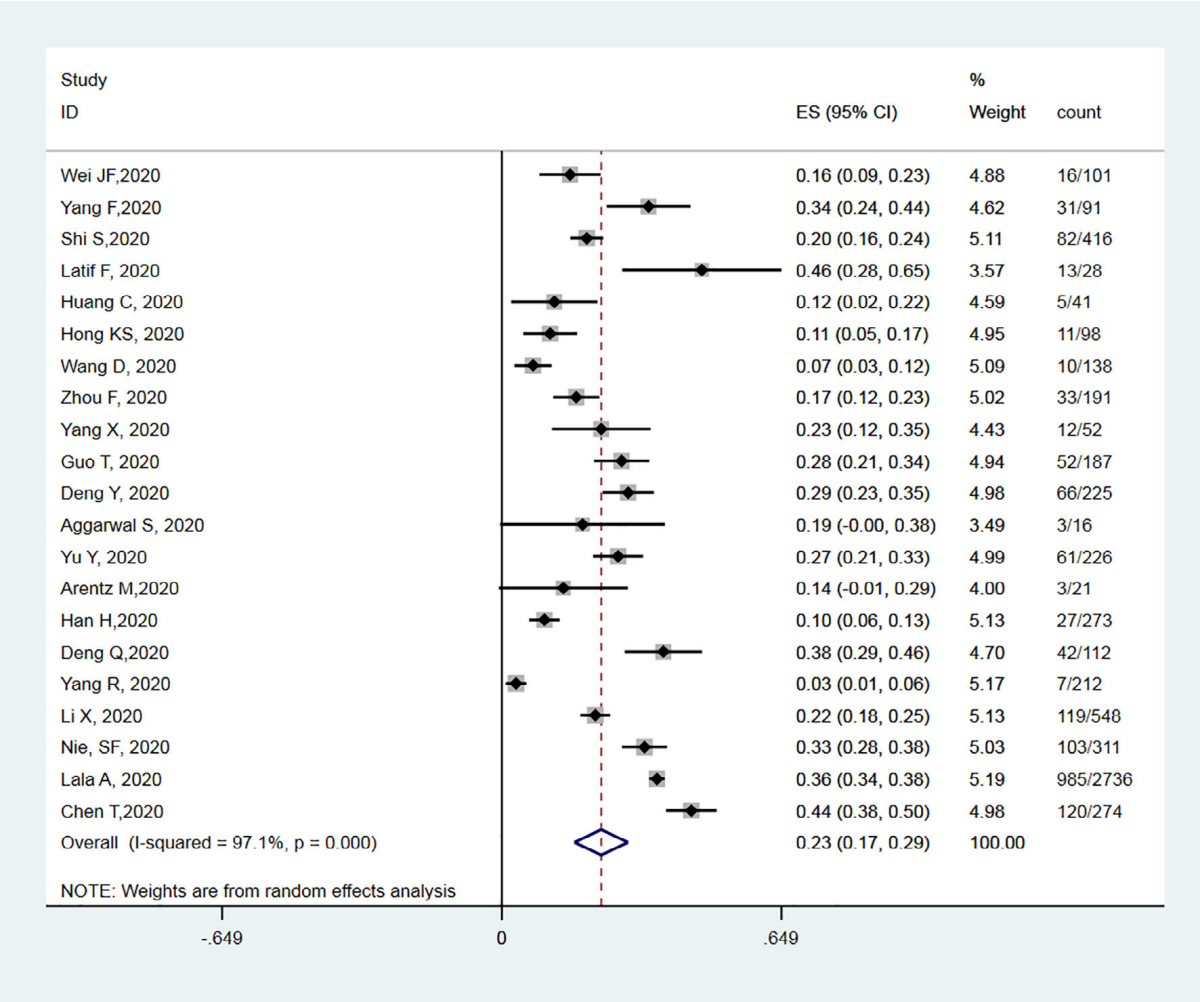
Meta‐analysis for the proportion of cardiac injury in patients hospitalized with COVID‐19

As presented in Figure 1, a total of 20 studies^3^, ^4^, ^6^, ^7^, ^8^, ^9^, ^10^, ^11^, ^12^, ^13^, ^14^, ^15^, ^16^, ^17^, ^18^, ^19^, ^20^, ^21^, ^22^, ^23^ comprising 6130 hospitalized patients with COVID‐19 were included in the meta‐analysis, resulting a pooled cardiac injury prevalence of 22% (95% CI: 16% to 28%), with a high heterogeneity (I^2^ = 97.0%).

**FIGURE 2:**
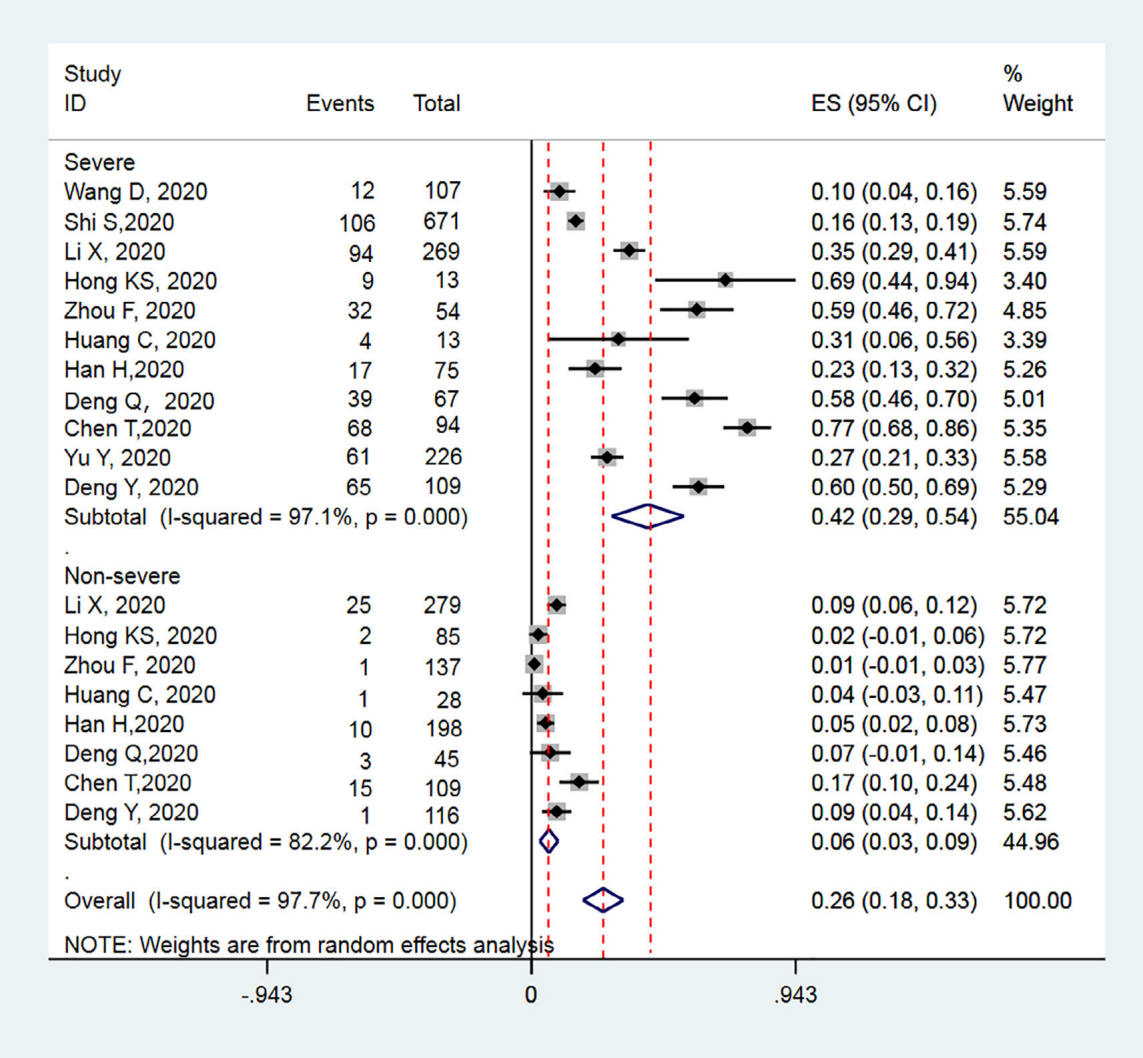
Meta‐analysis for the proportion of cardiac injury in patients hospitalized with severe and non‐severe COVID‐19

There were 11 studies reported the incidence of cardiac injury in severe COVID‐19 and non‐severe COVID‐19 patients, with 1781 or 914 subjects, respectively. The overall incidence of cardiac injury ranges from 10.2% to 69.2% and 1.0% to 9.0% in severe and non‐severe COVID‐19 patients, respectively. The pooled prevalence of cardiac injury was 42.0% (95% CI: 29% to 54%, I^2^ = 97%) among severe patients with COVID‐19 and 6% (95% CI: 3% to 9%, I^2^ = 77%) among non‐severe patients with COVID‐19 with a high heterogeneity (Figure 2).

In further subgroup analysis defined by age, there was a significant increased incidence of cardiac injury in older COVID‐19 patients (≥60 years) (ES: 15%, 95% CI: 10% to 21%, I^2^ = 92%) compared with young COVID‐19 patients (ES: 30%, 95% CI: 25% to 36%, I^2^ = 92%) (< 60 years) (Figure S2). There was no significant difference in cardiac injury in patients with COVID‐19 among the region subgroup (China and USA) (Figure S3).

### 3.4 The impact of cardiac injury on all‐cause death in hospitalized patients with COVID‐19

**FIGURE 3:**
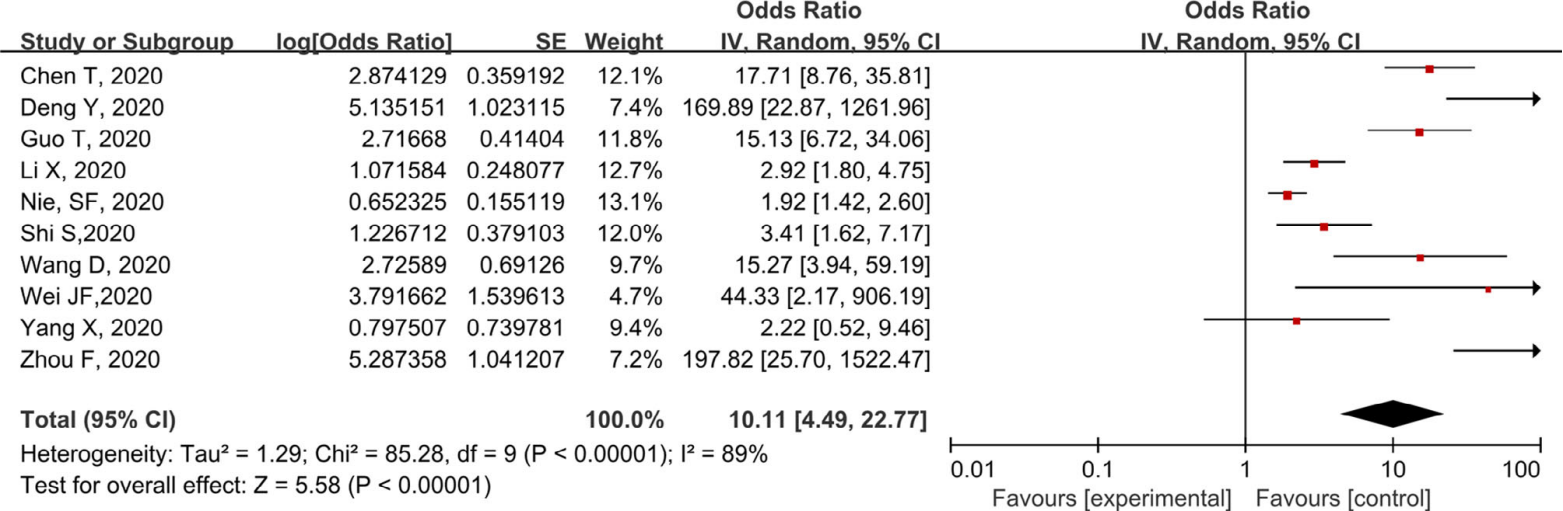
Meta‐analysis for association between cardiac injury and all‐cause death in patients hospitalized with COVID‐19

There were 10 publications^3^, ^4^, ^12^, ^13^, ^19^, ^20^, ^21^, ^22^, ^23^, ^24^ reported the association between cardiac injury and death. As shown in Figure 3, cardiac injury is associated with an increased risk of all‐cause mortality in patients with COVID‐19 (OR: 10.11, 95% CI: 4.49–22.77; I^2^ = 89%). For three studies^13^, ^20^, ^21^ that provided risk estimates adjusted for clinical confounding, the result did not change, with summary OR for 2.43 (95% CI: 1.71–3.46, I^2^ = 40%).

### 3.5 The impact of cardiac injury on all‐cause death in hospitalized patients with severe COVID‐19

**FIGURE 4:**
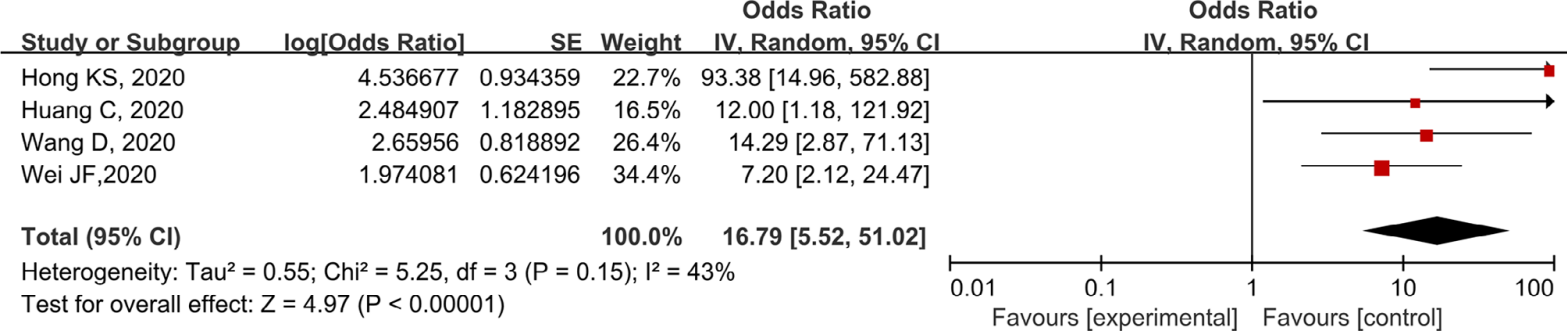
Meta‐analysis for association between cardiac injury and all‐cause death in patients hospitalized with severe COVID‐19 cases

There were two studies^4^, ^7^, ^12^, ^15^ reported the association between cardiac injury and death in patients with severe COVID‐19. None of included studies reported the adjusted results, thus, forced us to perform a univariate analysis. As presented in Figure 4, cardiac injury is associated with an increased risk of all‐cause mortality in patients with severe COVID‐19 (OR: 16.79, 95% CI: 5.52–51.02; I^2^ = 43%).

## 4 DISCUSSION

To our knowledge, this was the first meta‐analysis exploring the prevalence and impact of cardiac injury on COVID‐19. A total of 21 studies^3^, ^4^, ^6^, ^7^, ^8^, ^9^, ^10^, ^11^, ^12^, ^13^, ^14^, ^15^, ^16^, ^17^, ^18^, ^19^, ^20^, ^21^, ^22^, ^23^, ^24^ including 7076 COVID‐19 patients were selected and assessed. Our analysis indicated that cardiac injury is commonly observed in hospitalized patients with COVID‐19, especially in severe patients. Moreover, cardiac injury was associated with higher risk of all‐cause mortality in hospitalized patients with COVID‐19.

COVID‐19 is a systemic illness characterized by hyperinflammation and cytokine storm. The close association between COVID‐19 and cardiovascular disease has been observed in many studies. Cardiovascular disease, such as hypertension, hyperlipidemia, and coronary heart disease, are the common comorbidity in patients with COVID‐19. Those patients with cardiovascular comorbidity are at increased risk of morbidity and mortality. Those cardiovascular co‐morbidities may be related to cardiac injury in COVID‐19. Recent evidence^33^ also showed that COVID‐19 can damage the heart directly. Consistently, our multivariable analyses showed that elevated troponin release is independently predicted all‐cause death after adjusted for clinical confounding, including cardiac cardiovascular co‐morbidity. Cardiovascular manifestations, such as myocarditis, heart failure, arrhythmia, may occur with COVID‐19. More recently, there is increasing awareness of the cardiac injury in patients with COVID‐19 disease.

As we found, up to 23% of hospitalized patients with COVID‐19 have cardiac injury. The exact mechanisms of cardiac injury in COVID‐19 are not fully understood. Several potential pathogenic mechanisms appear to be involved. One potential mechanism is directly involves viral infiltration into myocardial tissue, resulting in cardiomyocyte death and inflammation.^34^ Another proposed mechanisms of COVID‐19–related cardiac injury include a cytokine storm manifested by elevated levels of interleukin‐6, ferritin, lactate dehydrogenase, and D‐dimer, which mediated by an imbalanced response by type 1 and type 2T helper cell.^3^ Increased cytokines and chemokines have been reported in patients with COVID‐19 who are admitted to an ICU as compared to non‐ICU patients,^15^ which may explain the higher cardiac injury in patients requiring intensive care unit care. Other suggested mechanisms include cardiac myocyte apoptosis due to hypoxia‐induced excessive intracellular calcium and cardiac stress lead by respiratory failure and hypoxemia.^35^

Age have been identified as an important risk factor for poor prognosis in patients with covid‐19. Our results also showed advance older patients (>60 years) with COVID‐19 have a higher prevalence rate of cardiac damage compared with young people. This founding was not surprising, as we all known, advance age higher rates of underlying CVD and other comorbid conditions, including hypertension, coronary heart disease, cardiomyopathy. Thus, these patients might more prone to have elevated TnT levels and experience myocardial injury during the course of COVID‐19. Sex was also a Lala study reported myocardial injury was more frequent compared to reports from China. We demonstrate that myocardial injury was not difference between the China and USA. However, the studies in USA is limited, the extract prevalence of cardiac injury in USA need further investigated. There might be a sex difference in the prevalence of cardiac injury in COVID‐19. Sex is a common modified factor in the risk and outcomes of CVDs^36^. Some evidence also showed that there might be a sex predisposition to COVID‐19, with men more prone to being affected.^37^ However, there were limited studies assessed whether there is a sex difference in the prevalence of cardiac injury in COVID‐19. Guo’ cohort^22^ showed male was more prone to experience an elevated TnT level (*p* = .005). In contract, another study from china did not find the sex difference (*p* = .39). Therefore, the sex difference in the risk of cardiac injury in COVID‐19 is still not clear and needed further studied.

Moreover, severe COVID‐19 patients (42%) have up to seven‐fold prevalence cardiac injury compared with the non‐severe COVID‐19 patients (6%). These results suggested COVID‐19 patients with cardiac injury might experience a worse prognosis. This hypothesis was further supported by our prognosis analysis, that elevated serum troponin is associated with an increased risk of severity and all‐cause mortality in patients with COVID‐19. Similarly, our results also showed that cardiac injury is associated with poor prognosis. Myocardial injury are common and associated with higher risk of all‐cause mortality in hospitalized patients with COVID‐19. Moreover, it was notable that a few studies reported that a proportion of patients with underlying CVD but with normal TnT levels had a relatively favorable outcome. Therefore, the uses of serum troponin testing may be a potential tool to facilitate risk stratification, help make decisions among hospitalized COVID‐19 patients. These results might call out the patients with induction of cardiac injury, more attention, prioritized treatment and even more aggressive treatment strategies should be reasonable. Although up to date, until now, no specific antiviral drugs have been recommended for COVID‐19, Dexamethasone^38^ and Remdesivir^39^ might be applied. The dexamethasone was reported be able to reduce the death^38^ and the Remdesivir was showed to reduce the length of hospital stay.^39^

Elevated serum troponin concentrations are the hallmark of cardiac injury, including but not limited to myocardial infarction.^5^ The high prevalence of cardiac injury may increase the need for cardiology consultation. Interpretation of cardiac injury requires cautiously. In patients with COVID‐19, the phenotypes of myocardial injury in COVID‐19 include viral myocarditis, stress cardiomyopathy, tachyarrhythmia, coronary microvascular ischemia, pulmonary embolism, type 1 myocardial infarction, and type 2 myocardial infarction.^40^ The phenotypes of cardiac injury must be elucidated according to clinical scenarios. According to latest guideline, minority of patients with cardiac injury is diagnosed with acute myocardial infarction. In a case series of 18 patients with confirmed COVID‐19 who had ST‐segment elevation on electrocardiography, 10 patients underwent coronary angiography, of whom two thirds had nonobstructive disease.^41^ Hence, we need to take together with clinical information, electrocardiogram, cardiac echocardiography, coronary angiography, and cardiac MRI to assess the cause of cardiac injury.

### 4.1 Study limitations

Some limitations may influence the validity of this meta‐analysis. First, all the included studies were observational studies and some studies did not adjust for the clinical confounding in the outcome of death and these biases might influence on our results. For example, ACEI/ARB, as we describe previously, was showed be associated with decreased risk of death in COVID‐19^42^. However, although with number changes, the positive association between cardiac injury and death was still persisted when excluding the unadjusted studies, which showed the robustness of our conclusion. Second, the definition of the cardiac injury differed across the included trials and potentially affected the findings. Third, the majority of studies are from China, the studies involved with the prevalence and impact of cardiac injury on COVID‐19 is needing to confirm this conclusion. Fourth, high heterogeneity existed across studies in some comparisons, limiting the interpreting on the results, however, we attempted to account for this by using a random effect model to make our results more conservable. The precise number for the type of cardiac injury is lacking, therefore, we cannot evaluate the association between type of cardiac involvement and adverse prognosis. Finally, lots of studies did not provide the adjusted results, thus, further studies with well‐designed are needed to confirm our results.

## 5 CONCLUSIONS

This was the first meta‐analysis exploring the prevalence and impact of cardiac injury on COVID‐19. Cardiac injury is common in hospitalized patients and advanced age and severe COVID‐19 patients prone to experience more risk of cardiac injury. Furthermore, cardiac injury is associated with increased risk of all‐cause mortality.

## CONFLICT OF INTEREST

All authors have no conflicts of interest that might be relevant to the contents of this manuscript.

## Supporting information


**Supplementary Table 1** Electronic search strategies determined on July 2020
**Supplementary Table 2** The definition of cardiac injury
**Supplementary Table 3**. Joanna Briggs Institute critical appraisal checklist applied for included studies
**Supplementary Table 4**. Quality assessment of the included studies by Newcastle–Ottawa scale

**Fig. S1** Flow diagram of the study selection process

**Fig. S2** Meta‐analysis for the proportion of cardiac injury in patients hospitalized with COVID‐19, stratified by age.

**Fig. S3** Meta‐analysis for the proportion of cardiac injury in patients hospitalized with COVID‐19, stratified by region.
