# Supplementary material for: Prevalence and impact of cardiac injury on COVID‐19: A systematic review and meta‐analysis
Source: Clin Cardiol. 2020 Dec 31;44(2):276–83. doi: 10.1002/clc.23540 (PMC7852167; doi:10.1002/clc.23540)
Supplement: Supplementary file 1 — Supplementary Table 1 Electronic search strategies determined on July 2020 Supplementary Table 2 The definition of cardiac injury Supplementary Table 3. Joanna Briggs Institute critical appraisal checklist applied for included studies Supplementary Table 4. Quality assessment of the included studies by Newcastle–Ottawa scale [file CLC-44-276-s001.docx]

**Supplementary Table 1. Electronic search strategies determined on July 2020**

| **Search** | **Query** |
| --- | --- |
| **The Cochrane library** |  |
| #1 | 2019-novel coronavirus OR SARS-CoV-2 OR COVID-19 OR 2019-nCoV |
| #2 | cardiac injury OR myocardial injury OR cardiac troponin |
| #3 | #1 and #2 |
| **PubMed** |  |
| #1 | 2019-novel coronavirus [Mesh] OR 2019-novel coronavirus [Text Word] OR SARS-CoV-2 [Mesh] OR SARS-CoV-2 [Text Word] OR COVID-19 [Mesh] OR COVID-19 [Text Word] OR 2019-nCoV [Mesh] OR 2019-nCoV [Text Word] |
| #2 | cardiac injury [Mesh] OR cardiac injury [Text Word] OR myocardial injury [Mesh] OR myocardial injury [Text Word] OR cardiac troponin [Mesh] OR cardiac troponin [Text Word] |
| #3 | #1 and #2 |
| **Embase** |  |
| #1 | '2019-novel coronavirus '/exp OR SARS-CoV-2'/exp OR ' COVID-19'/exp OR ' 2019-nCoV '/exp |
| #2 | ' cardiac injury '/exp OR ' myocardial injury '/exp OR ' cardiac troponin '/exp |
| #3 | #1 and #2 |
| **MedRxiv** |  |
| #1 | 2019-novel coronavirus OR SARS-CoV-2 OR COVID-19 OR 2019-nCoV |
| #2 | cardiac injury OR myocardial injury OR cardiac troponin |
| #3 | #1 and #2 |

**Supplementary Table 2 The definition of cardiac injury**

| Studies | The definition of cardiac injury |
| --- | --- |
| Zhou F, 2020 | hs cTnI >28 pg/mL |
| Wang D, 2020 | cTnI level above the 99th percentile upper reference limit or new abnormal electrocardiography and echocardiography findings |
| Latif F, 2020 | hs cTnT >0.022 ng/m |
| Hong KS, 2020 | cTnI level above the 99th percentile upper reference limit or new abnormal electrocardiography and echocardiography finding |
| Arentz M, 2020 | Had troponin level >0.3 ng/mL, |
| Aggarwal S, 2020 | hs cTnT above the 99th percentile value or suggestive changes in echocardiogram |
| Yu Y, 2020(*Critical care*) | hs-TnI > 28 ng/L or TnI > 0.3 ng/mL |
| Yang F, 2020 | cTnI>0.04ng/ml |
| Wei JF, 2020 | hs- TnT>14 pg/ml |
| Li X, 2020 | hs-TnI > 15.6 pg/mL |
| Lala A, 2020 | cTnI >0.3 ng/mL |
| Huang C, 2020 | hs cTnI >0.028 ng/m |
| Han H, 2020 | Ultra‐TnI>0.04 |
| Deng Q, 2020 | cTnI >0.04 ng/mL, |
| Yang X, 2020 | hs cTnI >28 pg/mL |
| Wang D, 2020(*Critical care*) | Serum levels of cardiac biomarkers (eg, cTnI) were above the 99th percentile upper reference limit or new abnormalities were shown in electrocardiography and echocardiography. |
| Shi S, 2020 | cTnI>0.04ng/ml |
| Shi S, 2020(EHJ) | cTnI>0.04ng/ml |
| Nie SF, 2020 | cTnI with at least one value was above the 99th percentile upper reference limitduring hospitalization |
| Guo T, 2020 | cTnT were above the 99th percentile upper reference limit |
| Deng Y, 2020 | hs-TnI >28 pg/mL or new abnormalities shown on electrocardiography and echocardiography |
| Yang R, 2020 | Serum levels of cardiac biomarkers (e.g. troponin I) were above the 99th percentile upper reference limit or new abnormalities were shown in electrocardiography and echocardiography |

**Supplementary table 3. Joanna Briggs Institute critical appraisal checklist applied for included studies**

| Study (First Author, Year) | Sample was representative? | Participants appropriately recruited? | Sample size was adequate? | Study subjects and the setting described | Data analysis conducted | Objective, standard criteria, reliably used? | Appropriate statistical analysis used? | Confounding factors/ subgroups/ differences identified and accounted? | Subpopulations identified using objective criteria |
| --- | --- | --- | --- | --- | --- | --- | --- | --- | --- |
| Zhou F, 2020[^3^](#_ENREF_3) | Yes | Yes | Yes | Yes | Yes | Yes | Yes | Yes | Yes |
| Wang D, 2020[^4^](#_ENREF_4) | Yes | Yes | Yes | Yes | Yes | Yes | Yes | Yes | Yes |
| Latif F, 2020[^6^](#_ENREF_6) | Yes | Yes | No | Yes | Yes | Yes | Yes | Yes | No |
| Hong KS, 2020[^7^](#_ENREF_7) | Yes | Yes | Yes | Yes | Yes | Yes | Yes | Unclear | Yes |
| Arentz M, 2020[^8^](#_ENREF_8) | Yes | Yes | No | Yes | Yes | Yes | Yes | Unclear | No |
| Aggarwal S, 2020[^9^](#_ENREF_9) | Yes | Yes | Yes | Yes | Yes | Yes | Yes | Unclear | No |
| Yu Y, 2020[^10^](#_ENREF_10) | Yes | Yes | Yes | Yes | Yes | Yes | Yes | Unclear | Yes |
| Yang F, 2020[^11^](#_ENREF_11) | Yes | Yes | Yes | Yes | Yes | Yes | Yes | Yes | No |
| Wei JF, 2020[^12^](#_ENREF_12) | Yes | Yes | Yes | Yes | Yes | Yes | Yes | Yes | No |
| Li X, 2020[^13^](#_ENREF_13) | Yes | Yes | Yes | Yes | Yes | Yes | Yes | Yes | Yes |
| Lala A, 2020[^14^](#_ENREF_14) | Yes | Yes | Yes | Yes | Yes | Yes | Yes | Yes | Yes |
| Huang C, 2020[^15^](#_ENREF_15) | Yes | Yes | Yes | Yes | Yes | Yes | Yes | Yes | Yes |
| Han H, 2020[^16^](#_ENREF_16) | Yes | Yes | Yes | Yes | Yes | Yes | Yes | Yes | Yes |
| Deng Q, 2020[^17^](#_ENREF_17) | Yes | Yes | Yes | Yes | Yes | Yes | Yes | Yes | Yes |
| Yang R, 2020[^18^](#_ENREF_18) | Yes | Yes | Yes | Yes | Yes | Yes | Yes | Yes | No |
| Yang X, 2020[^19^](#_ENREF_19) | Yes | Yes | Yes | Yes | Yes | Yes | Yes | Yes | No |
| Shi S, 2020[^20^](#_ENREF_20) | Yes | Yes | Yes | Yes | Yes | Yes | Yes | Yes | Yes |
| Nie SF, 2020[^21^](#_ENREF_21) | Yes | Yes | Yes | Yes | Yes | Yes | Yes | Yes | No |
| Guo T, 2020[^22^](#_ENREF_22) | Yes | Yes | Yes | Yes | Yes | Yes | Yes | Yes | No |
| Deng Y, 2020[^23^](#_ENREF_23) | Yes | Yes | Yes | Yes | Yes | Yes | Yes | Unclear | Yes |
| Chen T, 2020[^24^](#_ENREF_24) | Yes | Yes | Yes | Yes | Yes | Yes | Yes | Yes | Yes |

**Supplementary table 4. Quality assessment of the included studies by Newcastle–Ottawa scale**

| Study (First Author, Year) | Selection | | | | | Comparability | Outcome | | | Total |
| --- | --- | --- | --- | --- | --- | --- | --- | --- | --- | --- |
|  | Exposed cohort | Nonexposed cohort | | Ascertainment of exposure | Outcome of interest |  | Assessment of outcome | Length of follow-up | Adequacy of follow-up |  |
| Zhou F, 2020[^3^](#_ENREF_3) | * | | * | * | * | * | * | * | * | 8 |
| Wang D, 2020[^4^](#_ENREF_4) | * | | * | * | * |  | * | * | * | 7 |
| Hong KS, 2020[^7^](#_ENREF_7) | * | | * | * | * |  | * | * |  | 6 |
| Wei JF, 2020[^12^](#_ENREF_12) | * | | * | * | * | * | * | * | * | 8 |
| Li X, 2020[^13^](#_ENREF_13) | * | | * | * | * | ** | * | * | * | 9 |
| Huang C, 2020[^15^](#_ENREF_15) | * | | * | * | * | * | * | * | * | 8 |
| Yang X, 2020[^19^](#_ENREF_19) | * | | * | * | * | * | * | * | * | 8 |
| Shi S, 2020[^20^](#_ENREF_20) | * | | * | * | * | ** | * | * | * | 9 |
| Nie SF, 2020[^21^](#_ENREF_21) | * | | * | * | * | * | * | * | * | 8 |
| Guo T, 2020[^22^](#_ENREF_22) | * | | * | * | * | * | * | * | * | 8 |
| Deng Y, 2020[^23^](#_ENREF_23) | * | | * | * | * |  | * | * |  | 6 |
| Chen T, 2020[^24^](#_ENREF_24) | * | | * | * | * | * | * | * | * | 8 |

**Figure S1**


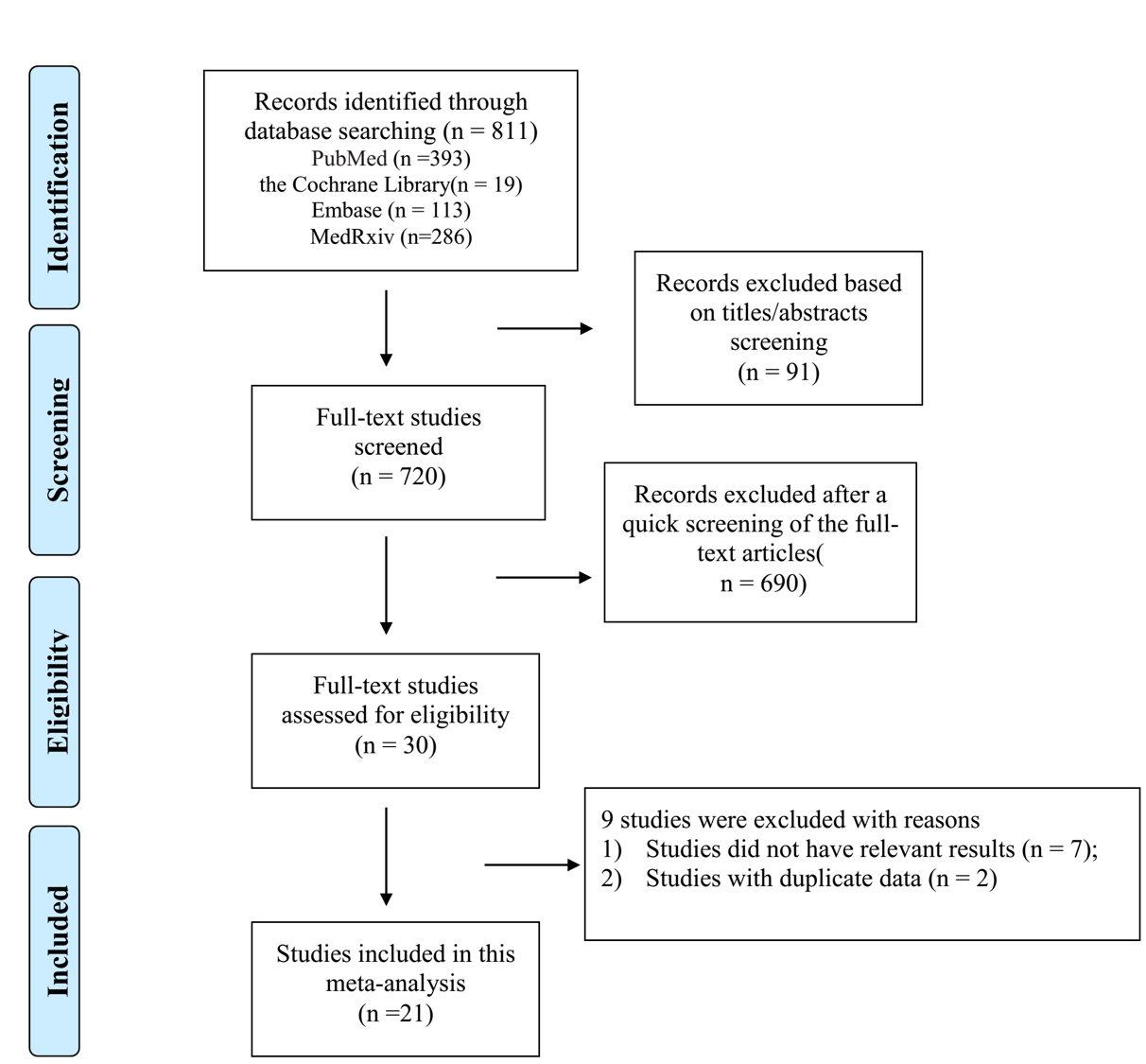


**Figure S2**

**
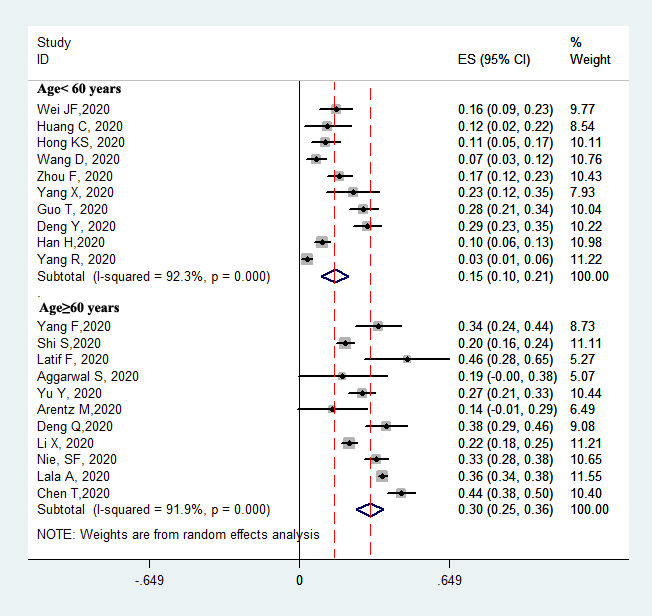
**

**Figure S3**

**
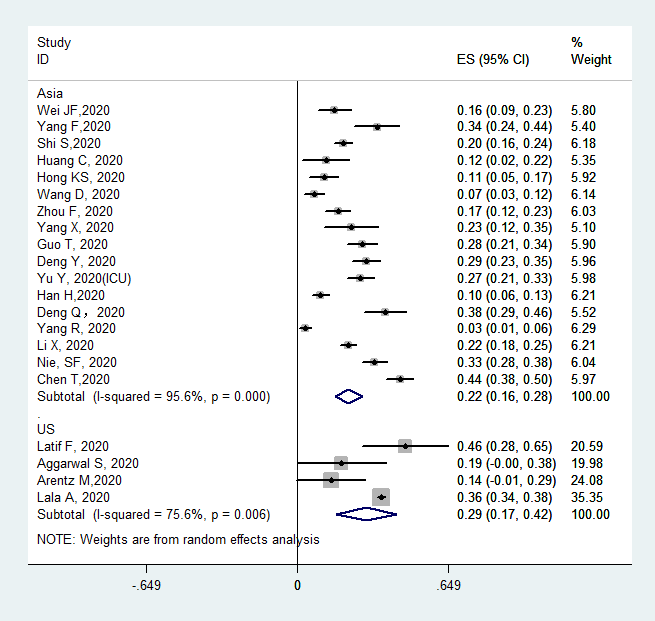
**
