# Supplementary figures and images for: Prevalence and impact of cardiac injury on COVID‐19: A systematic review and meta‐analysis
Source: Clin Cardiol. 2020 Dec 31;44(2):276–83. doi: 10.1002/clc.23540 (PMC7852167; doi:10.1002/clc.23540)

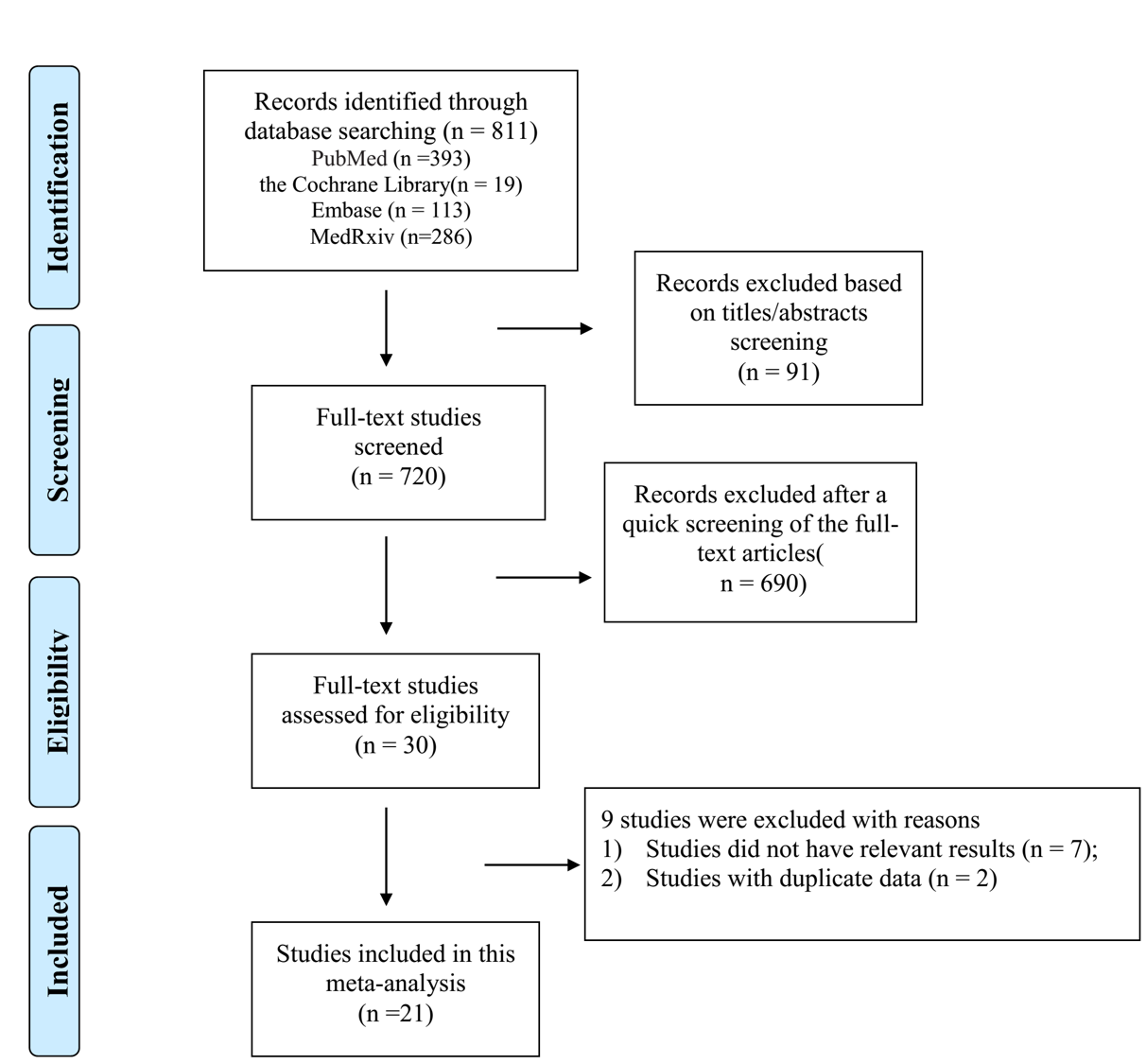

Supplement: Supplementary file 2 — Fig. S1 Flow diagram of the study selection process [file CLC-44-276-s002.tif]

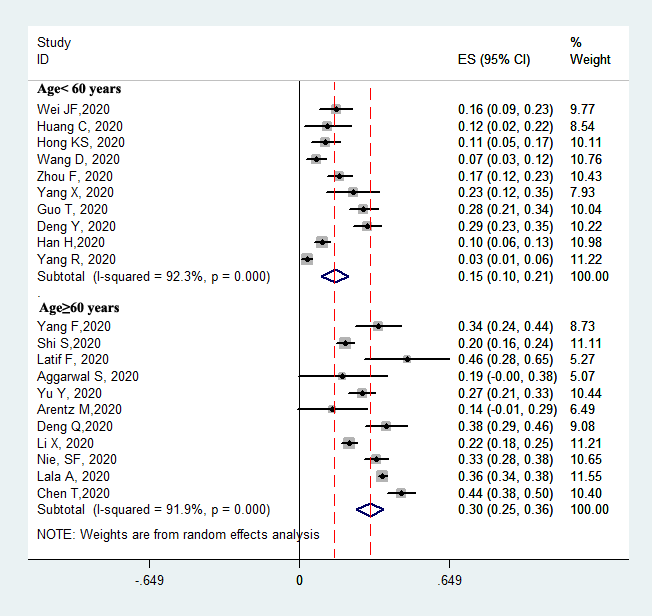

Supplement: Supplementary file 3 — Fig. S2 Meta‐analysis for the proportion of cardiac injury in patients hospitalized with COVID‐19, stratified by age. [file CLC-44-276-s003.tif]

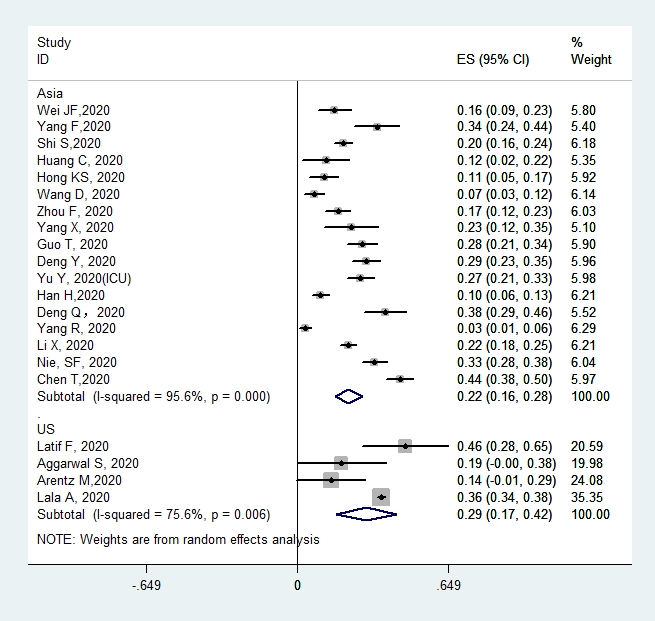

Supplement: Supplementary file 4 — Fig. S3 Meta‐analysis for the proportion of cardiac injury in patients hospitalized with COVID‐19, stratified by region. [file CLC-44-276-s004.tif]
